# Liver disease management as routine work in primary care: a qualitative interview study to guide implementation

**DOI:** 10.3399/BJGP.2022.0094

**Published:** 2022-10-18

**Authors:** Helen Jarvis, Tom Sanders, Barbara Hanratty

**Affiliations:** Population Health Sciences Institute, Faculty of Medical Sciences, Newcastle University, Newcastle upon Tyne.; Department of Social Work, Education and Community Wellbeing, Northumbria University, Newcastle upon Tyne.; Population Health Sciences Institute, Faculty of Medical Sciences, Newcastle University, Newcastle upon Tyne.

**Keywords:** attitude of health personnel, disease management, implementation science, liver diseases, primary health care, qualitative research

## Abstract

**Background:**

Morbidity from liver disease is rising in the UK. Most cases are caused by alcohol or non-alcoholic fatty liver disease (NAFLD) and treatable if caught early. Liver disease pathways have been shown to increase detection in the community, but have not been adopted into routine primary care work.

**Aim:**

To explore primary care healthcare professional (HCP) experiences and understanding of chronic liver disease, and where it might fit into management of long-term conditions.

**Design and setting:**

Qualitative interview study with 20 HCPs in primary care in the north of England.

**Method:**

A semi-structured approach informed by a theory of implementation (normalisation process theory [NPT]). Data collection and analysis were concurrent. Interview data were analysed using thematic analysis.

**Results:**

Participants identified the following key areas for action: incentivised frameworks and protocols to drive understanding, organise, and sustain practice; inclusion of common liver diseases into multimorbidity care to reduce complexity and workload; a need to define the GP role within a lifestyle-focused treatment pathway; and education/local champions to initiate and legitimise individual and organisational participation in change.

**Conclusion:**

To embed chronic liver disease management in routine primary care work, researchers and policymakers must be aware of the implementation challenges. These findings can guide the adoption of effective pathways and help bridge the implementation gap.

## INTRODUCTION

Morbidity and mortality from chronic liver disease is rising in the UK. It is a leading cause of premature mortality with an average age of death in the UK from liver disease of 57.^1^^,^^2^ Most cases of chronic liver disease are preventable and treatable if caught early and lifestyle interventions are enacted. Chronic damage to the liver is most commonly caused by excess alcohol, causing alcohol-related liver disease (ARLD), or obesity/metabolic risk factors leading to non-alcoholic fatty liver disease (NAFLD), or a combination of both. This increase in morbidity and mortality from liver disease contrasts sharply with decreases in the UK for other common long-term conditions.^3^ Currently around 70% of patients who present to accident and emergency departments with decompensated (end-stage) liver cirrhosis have had no previous diagnosis or management for their liver disease.^3^

In UK primary care there are well established long-term condition management pathways for diabetes, cardiovascular disease, and many other conditions. These evidence-based approaches are often run by the primary care nursing team, with oversight from primary care physicians. This work has gradually evolved under successive NHS contracts and reorganisation, initially encouraged under National Service Frameworks (NSFs) and subsequently incentivised under the Quality and Outcomes Framework (QoF) scheme.^4^ Introduced in 2004, the QoF is a system for the performance management and payment of GPs in the NHS.^4^

Chronic liver disease has been omitted from long-term condition management programmes in UK primary care and is not the subject of routine assessments or financial incentives. This is despite the fact that most annual reviews in primary care combine multiple long-term conditions within a single consultation, and liver disease shares risk factors with many other health problems. Primary care involvement in liver disease has generally been prompted by abnormal liver blood tests and focused on ruling out rare diseases and repeat testing. Guidance on appropriate response to risk factors and blood results, onward referral, or lifestyle interventions are inconsistent or absent. Several research studies have shown pathways to find chronic liver disease in the community lead to an increase in detection of significant disease^5^^,^^6^ and are cost-effective.^7^ Despite this, implementation of these pathways has been slow and partial^8^ and there has been little prospective study of how they may fit within routine primary care work.

This study explored primary care healthcare professional (HCP) experiences and understanding of chronic liver disease, and how this might fit into long-term condition management structures. This is part of a programme of work that aims to use implementation theory to inform the development of a framework to embed the management of chronic liver disease into routine primary care practice.

**Table:** How this fits in

Chronic liver disease is common but not actively managed in primary care. It is unclear how liver disease pathways could fit into routine work in primary care. This study highlights some of the challenges to implementing liver pathways and key areas for action. Clinicians identified the need for a defined role in an integrated and legitimised pathway, which should be part of multimorbidity care.

## METHOD

### Design

A qualitative cross-sectional study design used semi-structured interviews with HCPs working in primary care in the north of England (North East and North Cumbria). This study is reported in accordance with the standards for reporting qualitative research.^9^

### Recruitment

Participants were recruited from across the north of England using local GP and primary care commissioning networks. Invitations to participate were cascaded out to practices by email. Sampling of responders was purposive to allow for a variety of perspectives from HCPs working in demographically different practices with varying levels of experience. Experiences of primary care nurses and healthcare assistants as well as GPs were sought.

### Data collection

One author (a GP with expertise in liver disease in the community) conducted all the interviews via Zoom from October 2020 to May 2021. Interviews were digitally recorded, transcribed verbatim by a professional transcription company, and anonymised. Topic guides (Supplementary Document S1) were developed with reference to previous research with input from the wider project multidisciplinary group, including patient and public involvement (PPI) representatives. To provide an overall focus, while still allowing for flexibility, a semi-structured approach informed by a theory of implementation (normalisation process theory [NPT]) was used.

NPT is a middle-range implementation theory addressing factors needed for successful implementation and integration of interventions into routine work (normalisation).^10^^,^^11^ It is divided into four constructs:

- Coherence: what is the work that people do to understand and make sense of a practice?
- Cognitive participation: what is the work that people do to engage and support a new practice?
- Collective action: what is the work that people do to enact a new practice, and make it workable and integrate it in context?
- Reflexive monitoring: what is the work that people do to reflect on and evaluate enacting a new practice in context?

As the aim of this study was to inform intervention development, the first two constructs were most relevant to topic guide development, although data collection remained flexible to the dynamic nature of these constructs and consideration of the wider context.^12^

The topic guide was modified in response to early interviews, as the data collection progressed. Data collection continued until it was judged that sufficient data had been collected with no new depth or complexity arising from the interviews.

### Data analysis

Data collection and analysis were concurrent, with analysis starting as soon as the interviews were transcribed. Interview data were analysed using thematic analysis applying principles of constant comparison.^13^ The NVivo (version 12) software package was used to manage the data for coding. Although NPT had been used to inform the topic guide and ensure data on the relevant issues were collected, an inductive approach to analysis was employed. This approach gave participants the flexibility to raise issues important to them, and did not constrain them to NPT categories. Each transcript was coded by the author who conducted the interviews. All transcripts were independently analysed by at least two authors, with regular discussions among paper authors to refine developing themes. A final set of themes was agreed on by all co-authors. In a second step of analysis the themes were interpreted using the first two constructs of NPT.

### Patient and public involvement

This study sits inside a wider work programme of work, which has had significant PPI (both patients with chronic liver disease and representatives from liver charities).

## RESULTS

Twenty interviews were conducted online with HCPs working in primary care in the North East and North Cumbria region of England. Participants were GPs (*n* = 13) and members of the nursing team (*n* = 7), including nurse practitioners, practice nurses, and healthcare assistants. Interviews lasted 30–60 minutes. Demographic information is presented in Table 1. The list size distribution, profession, and experience level of staff and other documented demographics broadly fit with these distributions across UK general practice.^14^

**Table 1.** Participant demographic characteristics (*N* = 20)

**Interviewee characteristics**		***n***
**Sex**	Female	13
	Male	7

**Role**	GP	13
	Nursing team	7

**Experience in current role**	<5 years	6
	5–10 years	7
	>10 years	7

**Interest in liver disease^a^**	yes	2
	no	18

**Size of practice, number of registered patients**	<5000	4
	5000–10 000	10
	10 001–15 000	2
	>15 000	4

**Practice setting**	rural	6
	urban	10
	mixed/suburban	4

**Practice demographics**	deprived	8
	affluent	2
	mixed	10

a *Self-defined.*

Four themes that encapsulate the interviewees’ views and perceptions are presented:

- structural barriers to operationalising liver disease care;
- liver disease as part of multimorbidity;
- the value in managing liver disease; and
- facilitators of change in liver disease care.

The quotes illustrate themes that came out of many interviews while also highlighting any outlying views.

### Theme 1: Organisational barriers to care

Participants acknowledged that the two commonest causes of liver disease (ARLD and NAFLD) were ‘chronic’ in the sense that they required long-term management rather than acute treatment. Knowledge of the common preventable risk factors for liver disease was high. Despite this, the majority of participants shared the view that liver disease was not currently managed in the same standardised way as other long-term conditions within primary care. Reasons for this difference in approach to care for people at risk of liver disease were cited as primarily related to the organisational context and drivers of care, rather than individual clinical sense making.

To be considered a chronic disease in the primary care management context, it was felt that liver disease needed to be subject to protocols, with clear templates and guidelines. The primary care role was seen as being to implement and operationalise expertise brought together by others in clear guidelines, rather than to act independently to make clinical decisions outside of these protocols:

*‘The difficulty that I certainly find is that I never know — there’s not a clear protocol. If you think like with diabetes you know what you have to achieve. You know what you’ve got to aim for your blood pressure, you know what your HbA1c should be, you know what urine sample should be, you know what the cholesterol should be, so there’s very clear guidelines. With livers I think there’s difficulty knowing when it’s considered abnormal enough for investigation, what you then do with the results. When do you refer for a fibro scan, when is a fibro scan result important enough to need — it’s a very woolly area which I think if it was clear guidelines that told you, ‘’This is when you do x, y, and z.’’ Again, I think it could fall into more of a streamlined chronic disease model.’*

(GP4, GP partner 18 years, cancer lead, list size = 9600 mixed/semi-urban)

The absence of QoF incentives for liver disease was highlighted. Participants pointed to the importance of systemic and IT changes that accompanied QoF, rather than financial incentives. These triggered processes for a comprehensive structured approach to management, and provided a prompt to remind them to take action in a given area:

*‘I think QoF is useful for concentrating the mind. I think it’s never been a major driver in our practice. However, because the computer systems alerts and clever searches are often driven by QoF, I think things being on QoF benefit. So, for example, in diabetes when recording microalbuminurea came off QoF the figures dropped from 80% to 60% and I don’t think that’s anyone deliberately saying, ‘’Oh we’re not getting paid for this now so we’re not going to do it.’’ It’s about there weren’t alerts on the computers and everything else.’*

(GP5, GP partner 35 years, diabetes lead, list size = 9500 deprived/urban)

### Theme 2: Liver disease as part of multimorbidity

Some participants felt that liver disease was too complex to fit into a more structured clinical management approach. This perception arose in part from the custom of considering *all* liver disease as a diagnostic conundrum based on abnormal liver blood tests. When liver disease was framed in this context, participants were unable to see the relevance of other protocolised long-term condition care. As a result, active management of liver disease was more likely to be neglected:

*‘I think often a diagnosis as such of the liver disease is not made. So we get abnormal liver function tests for example and the response to that will quite often be simply to repeat the liver function tests after three months and then after six months and some people seem to get that continually and you look sort of two years down the line and they might not have had a liver screen done, so yeah I think it perhaps isn’t as well managed as some of the other conditions both in terms of the diagnosis but — and the response is often to repeat the blood tests rather than to necessarily get the patient in and ask about alcohol, lifestyle, check a BMI* [body mass index] *and those sorts of things.’*

(GP11, salaried GP 7 years, list size = 4000 mixed/rural)

Where participants considered the common lifestyle-related chronic liver diseases (NAFLD and ARLD) as separate from the other rare liver diseases, it was easier for them to see the sense in a more integrated, structured proactive approach. NAFLD and ARLD shared common risk factors with long-term conditions already being managed in primary care. This was seen as key by the majority of participants.

On a practical level it made sense to participants for liver disease to sit alongside other chronic diseases and be considered as part of multiple long-term condition care. Emphasising the impact that lifestyle advice could have on the liver, as well as other conditions, was perceived as helpful:

*‘I think it’s almost easier in a way because you say there’s too much fat in your liver and I think people have a visual — can see that, can think what does that look like more easily than what does diabetes mean? Or what does high blood pressure mean? I think that’s a really strong image for patients and they can see they’re too fat and then there’s fat in their liver …’*

(GP7, salaried GP 6 years, list size = 10000 mixed/semi-urban)

When participants considered embedding liver disease within existing structures for managing multimorbidity, they claimed that this would help to contain the workload. This was crucial when considering taking on new pathways of care:

*‘No, I think it would be quite easily encompassed in the screening because obviously we’re doing bloods anyway so potentially we’ll be looking at adding in a couple more bloods and obviously we’d be looking at patient’s BMI and other sort of risk factors so I don’t think potentially it would make a huge difference in the workload …’*

(Nurse [N]3, practice nurse 6 years, list size = 3500 mixed/rural)

### Theme 3: Seeing value by professional role

The perceived value of identifying and managing liver disease seemed to relate to professional role. Nurses’ positive approach to prevention and lifestyle interventions as treatment contrasted with the views expressed by some of the GPs. Doctors were more likely to link the value of liver disease management to the expectation of more ‘medical’ treatment. This tension led to some GPs struggling to identify their role within liver disease management and assuming, incorrectly, that other team members would not see beyond traditional doctor/patient expectations of a ‘treatment’:

*‘I guess the reason is because I don’t perceive an active treatment or benefit from monitoring. You know, they come back, and their ALT* [alanine transaminase] *is a bit worse next year. What am I going to do? Speak to them again and say, ‘’You didn’t really try hard enough with your diet? Are you still eating too much sugar?’’ or ‘’I think you’re lying to me about alcohol.’’ I don’t know. Awkward, awkward conversations’.*

(GP2, GP partner, 21 years list size = 11500 mixed/urban)

In contrast, the nurse participants felt that a liver pathway in chronic disease management would fit well into their current ways of working. They saw this as an extension of their established roles and expressed a willingness to be involved:

*‘I think if you can explain the fatty liver as a disease and what’s causing it and why they need to change their lifestyle they’re much more likely to engage with that. In a similar way to high blood pressure and diabetes, if you can really explain the relationships between these things and potentially they then see the results, so it’s actually really satisfying for people if they can actually reduce their BMI and their liver function gets better for example or their HbA1c comes down, they can actually see that effort paying off …’*

(N3, practice nurse 6 years, list size = 3500 mixed/rural)

### Theme 4: Facilitators of change in liver disease care

Education, legitimisation, and a local champion were seen as key facilitators to changing liver disease care in the community. Education gave practitioners confidence and allowed them to see the value of the intervention. This was noted as particularly important for the nurse participants to have effective and informed discussions with patients, despite not having been prioritised in any practice nursing curricula:

*‘We talk about alcohol and diet and things like that and it would be good to have some information to talk about liver disease for these certain patients so we can prevent things like that at first point instead of managing the condition later but no, we definitely don’t really talk about anything like that to be honest. I’ve seen it on patients’ notes but not been trained on it or anything. No.’*

(Healthcare assistant [HCA]1, healthcare assistant 6 years, list size = 5000 mixed/urban)

Prioritisation of a condition for inclusion in the QoF legitimised its importance and the need to change practice in that area. Participants gave this more weight than local pathways, as there was a perception that decisions made at national level had been through rigorous processes with more robust clinical reasoning from central decision makers. Such legitimisation was felt to be crucial to developing a common understanding among the whole practice team:

*‘Well, I think the whole point of it is its quality isn’t it? It’s not just the payment for it, it’s also that it’s seen at a national level that it’s important enough to go onto QoF. I think also in terms of getting practice managers engaged in the process as well and having it more as a wider team. I think if you were going to put this down as a diagnosis you’d want to retrospectively perhaps look at your patients to make sure you had everybody who had fatty liver disease on the register. It’s far easier to do that if you’ve got the practice management team on board and QoF definitely helps with that …’*

(GP11, salaried GP 7 years, list size = 4000 mixed/rural)

Participants stated that the importance they would attach to making liver disease a priority would also be enhanced by local colleagues within commissioning and secondary care. Someone championing change in an area of practice could make a lasting difference, and if this came from an ‘expert’ that was further evidence of the value of change:

*‘I think something like this which is probably quite a large-scale change in how we do things, I think probably we’d need somebody dedicated from the secondary care like gastroenterology setting who would actually perhaps work with some GPs who are particularly interested in the subject and develop a protocol between primary and secondary care that could be sent out to practices and adopted from there.’*

(GP4, GP partner 18 years, cancer lead, list size = 9600 mixed/semi-urban)

### Interpreting the findings using NPT

Although the themes were not constrained by NPT, as this action-based theory of implementation was used to guide the study process, in a second analysis step the themes were interpreted with reference to NPT. Table 2 summarises the themes presented and how primarily the first two constructs of NPT, coherence, and cognitive participation, can be used to help interpret these themes and provide a focus towards the work that individuals and organisations would need to do to enable chronic liver disease management to become a normalised part of long-term condition care.

**Table 2.** Mapping themes onto constructs of NPT

**Theme**	**Description of theme**	**Construct of NPT**	**Key area for action**
Organisational barriers to care	HCPs describe views on liver disease being part of routine chronic disease management: lack of framework/protocols lack of QoF	**Coherence:** differentiation (difference from other routine practice) communal specification (shared understanding) **Cognitive participation:** enrolment (organising to collectively contribute) activation (actions to sustain practice)	Standardised protocols/frameworks
Liver disease as part of multimorbidity	Understanding liver disease as part of long-term multicondition care: complexity separating NAFLD/ARLD workload	**Coherence:** differentiation individual specification (individual sense making) **Cognitive participation:** activation	Work to incorporate common liver diseases into multimorbidity care
Seeing value by professional role	HCPs assign value related to how treatment is perceived and role: seeing value in lifestyle interventions role of GP (unclear) versus nursing team (clear)	**Coherence:** individual specification, internalisation (understanding the value)	Define a clear role for GPs in liver disease care
Facilitators of change in liver disease care	HCPs’ views on what would initiate and maintain change: education legitimisation local champions	**Coherence:** internalisation **Cognitive participation:** initiation (making things happen) legitimisation (right to be involved)	Promote education and local/national champions

*ARLD = alcohol-related liver disease. HCP = healthcare professional. NAFLD = non-alcoholic fatty liver disease. NPT = normalisation process theory. QoF = Quality and Outcomes Framework.*

## DISCUSSION

### Summary

HCPs identified the lack of frameworks as a barrier to managing liver disease in a similar way to other chronic diseases. National frameworks such as QoF were seen to legitimise need and drive protocol development. Considering liver diseases as part of multimorbidity was identified as a way of reducing complexity, and minimising the workload of adding liver disease to long-term condition care. The value of earlier detection was accepted by the nursing team but not by all GPs. Education and legitimisation were found to be important facilitators to the change necessary to make liver disease management routine in primary care. By analysing the results with reference to an action-based implementation theory (NPT) insight has been gained into the work that organisations and individuals may need to do to develop a framework for managing liver disease effectively in primary care. As this research is happening at the development stage of implementing an intervention, these findings fall mainly with the core constructs of coherence (sense-making work) and cognitive participation (relational work). To make sense of, and be able to build and sustain a new way of working in the area of liver disease, participants identified key areas for action: integrated and incentivised frameworks and protocols to drive communal understanding as well as organise and sustain practice; incorporating common liver diseases into multimorbidity care to reduce complexity and allow individual sense making as well as manage workload; defining the GP role within a predominantly lifestyle-focused treatment pathway for GPs to better understand the value in change; and education/local champions to help initiate and legitimise individual and organisational participation in change.

### Strengths and limitations

To the authors’ knowledge, this is one of the first qualitative interview studies to look at implementation of chronic liver disease management into primary care. Early detection of liver disease is high on the national and international hepatology agenda but this study is one of the first to give attention to the primary care perspective. The timing of this research is a strength, as it was conducted as part of the process of intervention development, rather than retrospectively identifying implementation barriers to a care pathway. Findings are therefore being taken forward directly to guide a local pathway implementation strategy. The validity of the study was strengthened by the use of an action-focused implementation theory (NPT). Participants were aware of the researcher’s professional background, which helped build rapport and a common understanding. However, it is acknowledged that this may have influenced the content of participants’ narratives.^15^^,^^16^ Limitations also include the possibility of selection bias, as participants who were willing to be interviewed may hold different views from those who were not. The interviews were conducted remotely rather than face to face as initially planned (owing to the coronavirus pandemic) and it is acknowledged that this may have influenced rapport and therefore data collected.

### Comparison with existing literature

Several pathways to manage liver disease in the community have been developed and piloted.^5^^,^^6^^,^^17^^,^^18^ Most are in the UK, and focused on short-term clinical outcomes such as the number of referrals to secondary care and new cases of liver disease detected. A retrospective study of 29 HCPs’ experiences of specialist nurse-led clinics for community-based detection of liver disease identified some similar findings on barriers and facilitators to implementation. For example, practitioners required clear guidelines and responsibilities, and in this way saw themselves as functionaries of others’ expertise.^19^ In other work, the patient perspective on incorporating liver disease screening into community care has been studied.^20^^,^^21^ Although the studies differed in patient groups eligible and tests offered, common themes around the utility of a positive test result to initiate lifestyle change by providing something concrete to work towards came across in both studies. These findings are closely aligned with the nurses in this study in seeing the value of making a diagnosis to prompt discussion and targets as part of lifestyle intervention.

Other studies have looked at the implementation of chronic disease management in primary care settings. A systematic review of factors influencing the implementation of chronic care models was dominated by work on diabetes pathways.^22^ Of the synthesised findings related to HCP experiences, many of these were in common with this study, particularly within the theme of preparing HCP for change. Education for primary care practitioners, seeing a reason or value in change, and the need for supportive leadership to legitimise change were all recurring themes in the literature around management of other chronic disease. These themes, in common with this current study, support the findings and strengthen the recommendations for change, although none of the studies synthesised were related to chronic liver disease.

### Implications for research and practice

The results of this study will be used directly to guide the development of a chronic liver disease framework being implemented into routine long-term condition management in North East England. Key recommendations for change are to standardise and integrate management protocols, incorporate liver disease into multimorbidity care, define a clear role for GPs, and promote education and local champions to drive these changes.

The study also adds to the literature on implementation science. The data-derived themes map well to the first two constructs of NPT, emphasising the validity and usefulness of this theory to guide and structure healthcare intervention implementation.

To make chronic liver disease management a routine part of primary care work, researchers and policymakers must be aware of the implementation challenges. These theory-driven findings can guide the adoption of effective pathways and help bridge the gap between research findings and real-world intervention success.
